# Photochemically Aided Arteriovenous Fistula Creation to Accelerate Fistula Maturation

**DOI:** 10.3390/ijms24087571

**Published:** 2023-04-20

**Authors:** Yong He, Blake Anderson, Qiongyao Hu, RB Hayes, Kenji Huff, Jim Isaacson, Kevin S. Warner, Hank Hauser, Myles Greenberg, Venita Chandra, Katalin Kauser, Scott A. Berceli

**Affiliations:** 1 Division of Vascular Surgery and Endovascular Therapy, Department of Surgery, University of Florida, Gainesville, FL 32611, USA; 2 Alucent Biomedical Inc., Salt Lake City, UT 84108, USA; 3 Division of Vascular Surgery, Department of Surgery, Stanford University, Stanford, CA 94305, USA; 4 North Florida/South Georgia Veterans Health System, Gainesville, FL 32608, USA

**Keywords:** hemodialysis vascular access, arteriovenous fistula maturation, natural vascular scaffolding, venous angioplasty, drug-coated balloon, 10-8-10 Dimer, extracellular matrix, photochemical treatment, photoactivated linking, outward vascular remodeling

## Abstract

Rates of arteriovenous fistula maturation failure are still high, especially when suboptimal size veins are used. During successful maturation, the vein undergoes lumen dilatation and medial thickening, adapting to the increased hemodynamic forces. The vascular extracellular matrix plays an important role in regulating these adaptive changes and may be a target for promoting fistula maturation. In this study, we tested whether a device-enabled photochemical treatment of the vein prior to fistula creation facilitates maturation. Sheep cephalic veins were treated using a balloon catheter coated by a photoactivatable molecule (10-8-10 Dimer) and carrying an internal light fiber. As a result of the photochemical reaction, new covalent bonds were created during light activation among oxidizable amino acids of the vein wall matrix proteins. The treated vein lumen diameter and media area became significantly larger than the contralateral control fistula vein at 1 week (*p* = 0.035 and *p* = 0.034, respectively). There was also a higher percentage of proliferating smooth muscle cells in the treated veins than in the control veins (*p* = 0.029), without noticeable intimal hyperplasia. To prepare for the clinical testing of this treatment, we performed balloon over-dilatation of isolated human veins and found that veins can tolerate up to 66% overstretch without notable histological damage.

## 1. Introduction

For patients with end-stage renal disease, hemodialysis is the most common therapy to maintain adequate electrolyte balance and remove metabolic waste products. Arteriovenous fistulas (AVFs), created through the surgical or percutaneous anastomosis of an artery and vein in the upper extremity, are the preferred form of vascular access for chronic hemodialysis. The Achilles heel of AVFs is that approximately half of them fail to mature and become clinically functional [1,2]. Ideally, an AVF’s venous luminal diameter increases progressively over the first several weeks after its creation until it becomes sufficiently large to deliver high blood flow to the extracorporeal circuit required for adequate dialysis. There is increasing evidence that the ability of the lumen to expand, either by functional dilatation, structural outward remodeling of the wall, or both, is a crucial determinant of the fistula’s luminal diameter and future fate [1,3,4,5].

The physiologic demands required for successful AVF maturation are unique in the spectrum of adaptive remodeling following a vascular intervention. After angioplasty, stenting, or vein bypass grafting, the primary therapeutic goal is to minimize intimal hyperplasia through a reduction in smooth muscle cell (SMC) proliferation and extracellular matrix (ECM) deposition. In contrast, successful AVF maturation requires both expansive remodeling and a dampened inward hyperplastic response. The difficulty in achieving this goal is the inherent opposition in the biological response needed to meet this desired phenotype. Specifically, expansive remodeling requires reorganization of the matrix accompanied by SMC detachment, migration, and proliferation, while the mitigation of occlusive hyperplasia requires inhibition of the SMC migration and proliferative response as well as control of ECM synthesis and deposition [6,7]. Although paclitaxel- or sirolimus-coated balloons have demonstrated efficacy in salvaging failed AVFs, conventional anti-proliferative therapies currently in clinical use for other vascular pathologies are not suitable to meet these unique demands [8].

**Figure 1:**
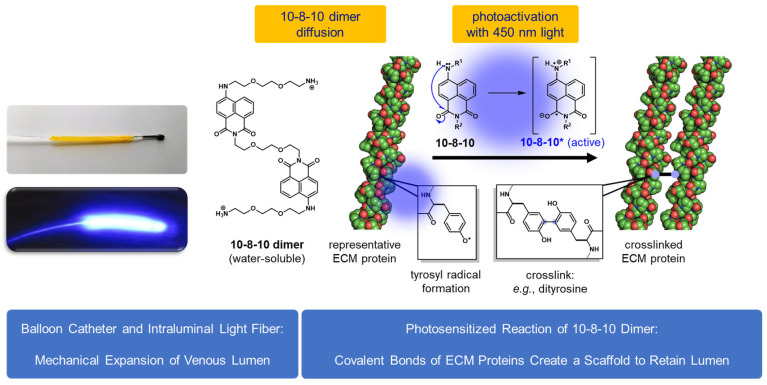
Mechanism of Action of the VRS-AVF Treatment. The Vascular Restoration System (VRS) delivers a photoactivatable small molecule (10-8-10 Dimer) to the vein using an intraluminally introduced balloon during surgery prior to arteriovenous fistula (AVF) creation. Light activation of the molecule with a 450 nm light at the time of balloon distension of the vein results in a photochemical reaction, facilitating new covalent bound formation among oxidizable amino acids of the extracellular matrix (ECM) in the vein wall at the site of the balloon. Creating this natural vascular scaffold around the extended lumen aims to mitigate the hemodynamic stress but allow outward smooth muscle proliferation for a faster maturation of the treated veins. PDB file used to make the collagen image is 1CAG. 1CAG rendered using The PyMOL Molecular Graphics System, Version 2.0 (Schrödinger, Inc., New York, NY, USA).

ECM proteins are in dynamic connectivity with the cellular components of the vascular wall [9,10,11]. Structural proteins connect with transmembrane proteins, such as integrins, to elicit signal transduction and mediate cellular responses driving the remodeling process of the vascular wall in response to hemodynamic changes [12,13]. Alucent Biomedical is focusing on targeting the vascular ECM as a therapeutic approach using a unique coated balloon device—called vessel restoration system for AVF creation (VRS-AVF)—to improve fistula maturation (Figure 1). Light activation of a small molecule 10-8-10 Dimer (polyetheramine-4-amino-1,8-naphtalamide) at the time of balloon inflation results in a photochemical reaction facilitating new covalent bond formation among oxidizable amino acids of the ECM proteins of the vein wall, retaining the size of the balloon distension [14,15,16]. Alteration in the ECM environment of cells regulates vascular responses, and it is responsible for adaptive or maladaptive remodeling of the vascular wall [17,18]. The ECM seems to play a critical role in directing cell proliferation to achieve outward remodeling [19,20]. Our hypothesis is that the newly formed covalent bonds within the ECM by the endovascular photochemical reaction of the VRS-AVF treatment support the balloon-dilatation-initiated lumen size and concomitantly allow the arterial pressure-induced proliferative response of the vein cells to assist outward remodeling.

Using a clinically relevant sheep model, the current study seeks to evaluate the impact of intraluminal balloon dilatation and photochemically induced covalent bond formation within the ECM on the kinetics of AVF remodeling. Specifically, the AVF morphology and cell kinetics at one and four weeks following the application of the VRS-AVF technology were examined. To set the stage for advancing this approach to a clinical trial, we utilized excised segments of human veins to define safe operating parameters for the system.

## 2. Results

### 2.1. Sheep AVF Maturation at 1 Week and 4 Weeks Following Surgery

There was no occlusion observed in the AVF veins harvested at 1 week post-surgery from three sheep using the histological images. Two of the six 4-week post-surgery sheep had occluded veins at one side; one was the VRS-AVF-treated vein, and the other was a control vein. Therefore, occlusion seemed not to relate specifically to the treatment but might be a result of the complexity to control perioperative hemostasis in sheep [21]. At the end of the 4-week period, Doppler ultrasound measurements were also performed to assess AVF patency in conscious standing animals prior to euthanasia. These measurements confirmed patency of the fistulas in the four animals. Estimation of volume flow in this model was notably challenging given the non-uniformity of the flow patterns and the associated inaccuracies of the algorithms used by duplex ultrasound scanners to convert centerline velocity and vessel diameter to total flow rates. As such, we felt that the volume flow rates collected were not reliable for quantitative comparison. The volume flow was 349 ± 401 (range: 70–933) mL/min and 183 ± 81 (range: 104–288) mL/min for treated and control AVFs, respectively. These flow rates were low compared with the flows typically observed in mature human AVFs, but they were not unexpected given the diameter and length of the inflow artery used to construct these fistulas.

One week after the surgery, the perivascular tissue exhibited seroma formation and other reactive soft tissue changes (early fibroplasia, inflammation, microvascular thrombosis, etc.) on both sides that were attributed to normal postoperative soft tissue healing. The VRS-AVF-treated veins exhibited increased compression injury, endothelial denudation, fibrin deposition, and inflammation compared with the untreated controls. However, all these changes were minimal to mild in severity.

**Figure 2:**
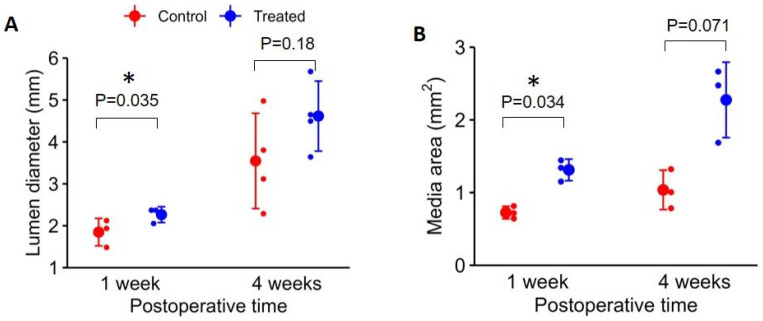
Histomorphometric Analysis of the Proximal Outflow Vein Sections from the Sheep AVF Studies. The illustrated histomorphometric analysis was performed on vein sections proximal to the anastomosis. (**A**) The size of the luminal diameter at 1 week and 4 weeks following surgery. The luminal diameter of the treated veins (blue dots) 1 week following surgery was significantly larger (*p* = 0.035) compared with control veins (red dots). The asterisks (*) in the graphs denote where statistically significant differences have been observed. By the 4th week following surgery, the difference was not statistically significant. (**B**) The corresponding media area of the same veins was also different at 1 week, the treated veins (blue dots) having a significantly (*p* = 0.034) thicker venous wall. This difference was not statistically significant by the 4th week. At 4 weeks, there were only three data points of media area because the artifacts in histological processing interfered with accurate quantification of the media area of one vein.

Our detailed morphologic study focused on the vein segments approximately 2 cm from the anastomosis because stenosis occurs most frequently at the swing portion of the vein, i.e., the vein segment that is dissected and moved to connect to the artery during surgical creation of an AVF. The mixed-effect model demonstrates that lumen diameters were significantly different between 1 week and 4 weeks (*p* = 0.0087, Figure 2A). The lumen diameter increased from 1 week to 4 weeks (lumen diameter for the treated vein: 2.26 ± 0.19 mm at 1 week and 4.62 ± 0.84 at 4 weeks; for the control vein: 1.85 ± 0.33 mm at 1 week and 3.55 ± 1.14 mm at 4 weeks). However, overall, the differences in vein diameters between the treated and control groups were not statistically significant (*p* = 0.065). Individual paired t-test showed that the treated vein lumen diameter was statistically significantly larger at 1 week (*p* = 0.035) but not at 4 weeks (*p* = 0.18) compared with the lumen diameter on the control side. There was no noticeable intimal hyperplasia in either treated or control veins. The mixed-effect model demonstrates that media areas were significantly different between 1 week and 4 weeks (*p* = 0.011, Figure 2B). The media area increased from 1 week to 4 weeks (for the treated vein: 1.31 ± 0.15 mm^2^ at 1 week and 2.27 ± 0.52 mm^2^ at 4 weeks; for the control vein: 0.73 ± 0.09 mm^2^ at 1 week and 1.04 ± 0.27 mm^2^ at 4 weeks). Overall, the differences in media areas between the treated and control groups also reached statistical significance (*p* = 0.0013). Individual paired t-tests showed that the treated vein media area was statistically significantly larger at 1 week (*p* = 0.034) but not at 4 weeks (*p* = 0.071).

### 2.2. Evidence of Facilitated Outward Remodeling Following VRS-AVF Treatment (Ki67 and SMC Staining)

**Figure 3:**
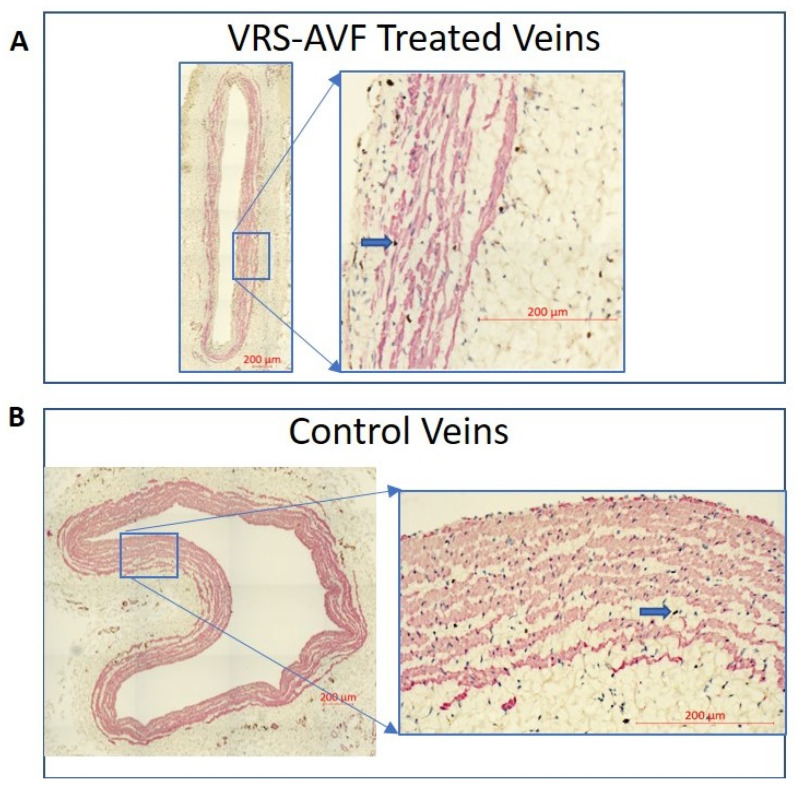
Evidence of Facilitated Outward Remodeling Following VRS-AVF Treatment (Ki67 and smooth muscle cell staining). Representative immunohistochemistry images of venous wall cross sections from fistulas collected 1 week following surgery. The upper panel (**A**) illustrates the VRS-AVF treated vein. Ki67 positive staining (dark brown dots, blue arrows) demonstrates the presence of more proliferating cells than can be seen in the control vein, lower panel (**B**). Smooth muscle actin (SMA) was stained red.

The expression levels of a molecular target of cell proliferation, Ki67, was selected for further investigation on the basis of the results from the morphometric analysis [22]. The mixed-effects model having treatment status and time demonstrated that the percentages of proliferating SMCs in treated veins were statistically significantly different from the control veins (*p* = 0.029). There were more proliferating SMCs in treated than control veins (Figure 3), but individual analysis at each timepoint failed to show a statistically significant difference on the basis of the paired t-tests (treated vs. control veins of the same animal) at 1 week (38 ± 21% vs. 11 ± 3% for treated and control veins, respectively) and 4 weeks (25 ± 14% vs. 12 ± 16% for treated and control veins, respectively), likely due to the small sample size and large variations.

### 2.3. Sheep vs. Human Vein Wall Size and Delivery of the 10-8-10 Dimer

**Figure 4:**
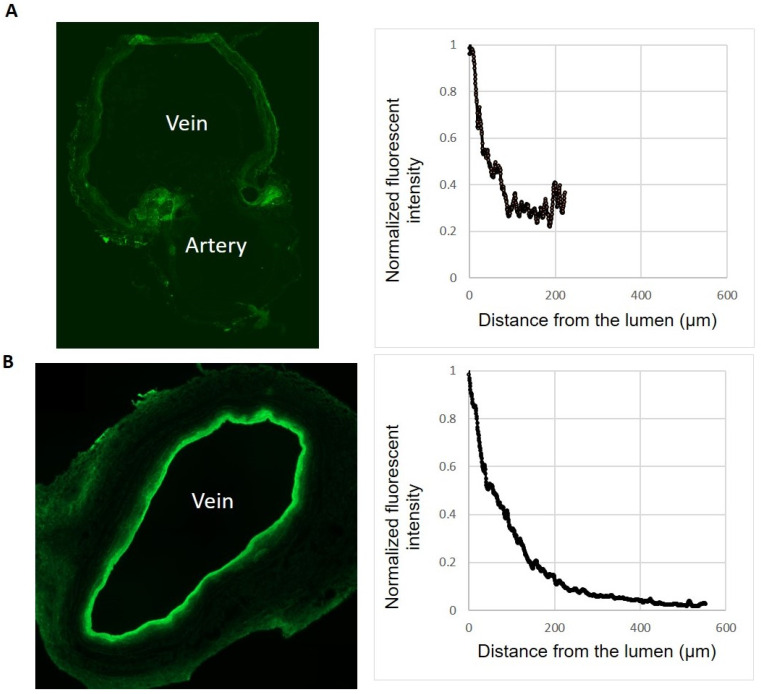
Human and Sheep Vein Differences. Fluorescent histological images of the cross section of sheep (**A**) and human (**B**) veins after VRS-AVF treatment. The fluorescent property of the 10-8-10 Dimer allows its detection in the vascular wall and helps monitor its distribution following treatment. The cross section of the sheep vein was taken from a freshly harvested vein immediately after VRS treatment and fistula creation surgery at the level of the anastomosis, including the feeding artery. The diagrams on the right help appreciate the significant wall thickness differences between sheep vein (**A**) and human vein (**B**) (X axis, distance from lumen/wall thickness) and the drug penetration into the wall (Y axis, fluorescent intensity normalized by the maximal intensity of each vein).

To apply the insights obtained from the sheep AVF study to human AVF, we need to consider the structural differences between sheep and human veins. Therefore, we compared the wall thickness (intima and media) of sheep veins harvested at the time of fistula creation (N = 3) and human arm veins (N = 6). The sheep veins were notably thinner than the human veins, but the difference due to the small sample size and large variations was not statistically significant (183 ± 91 vs. 386 ± 283 µm; *p* = 0.095). This difference in wall thickness can be appreciated from the fluorescent histological images of the cross sections collected immediately following the treatment (Figure 4). The distribution of the 10-8-10 Dimer can be visualized due to its fluorescent property. The human arm veins were treated ex vivo. As a result of the intraluminal application, the concentration in the vessel wall decreases away from the lumen.

### 2.4. Human Vein Distension and Biomechanics

**Figure 5:**
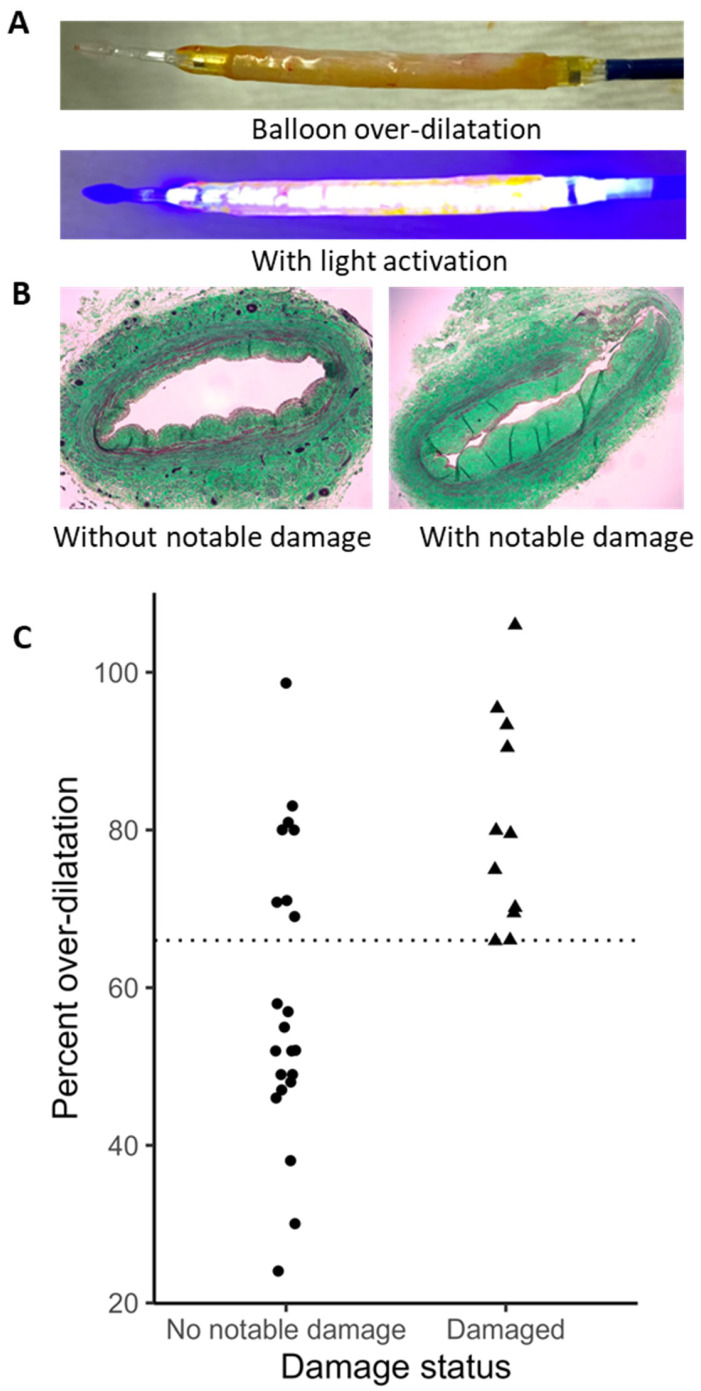
Human Vein Treatment with VRS-AVF Ex Vivo. Panel A demonstrates representative human vein segments cannulated and dilated by the VRS-AVF device. The yellowish color is a result of the 10-8-10 Dimer coat on the balloon, which diffuses into the venous wall during treatment. The lower picture of panel (**A**) shows the light activation phase during balloon dilatation at a distended lumen size. Panel (**B**) demonstrates two histological cross sections stained with Masson’s staining. The image on the left-hand side illustrates an intact vein, and the right-hand side illustrates a damaged vein histological cross section as a result of the treatment with balloon dilatation. Panel (**C**) summarizes results of all 33 human vein segments studied. The dotted line at 66% represents the maximum dilatation the vein can accommodate before damage of the vessel wall can be seen in histological images. Important to note that some veins resisted even 100% dilatation without visible damage.

**Figure 6:**
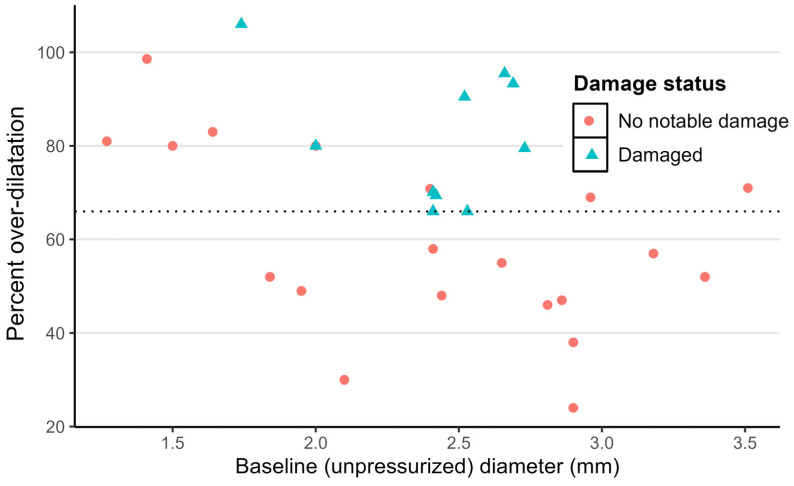
Distribution of Over-Dilatation by Baseline Diameter of Human Veins. Distribution of percent over-dilatation of human vein segments dilated by the VRS-AVF device. The X axis depicts the baseline lumen diameter of the veins prior to treatment. The Y axis shows the percent over-dilatation over the baseline. Each vein is represented by one data point. Red dots illustrate veins without any histological damage, and blue triangles represent veins with noticeable histological damage.

Thirty-three human vein segments obtained from amputated legs were treated with VRS-AVF under different levels of balloon over-dilatation to assess the relationship between the injury status and the level of overstretch. An example of the vein at the time of over-dilatation but before light activation is shown in the top panel of Figure 5A and during light activation in the lower panel of Figure 5A. Figure 5B shows representative histological sections of human veins without or with notable damage following VRS-AVF treatment. Twenty-two veins did not show discernable damage, but 11 veins had detectable damage or leakage following balloon overstretch. All damaged veins had at least 66% over-dilatation, although there was an example of no notable damage for a vein with approximately 100% over-dilatation (Figure 5C). The distribution of percent over-dilatation with the baseline diameter is shown in Figure 6. It is interesting to note that the four veins with at least 80% overstretch but without notable damage had a lumen diameter smaller than 2 mm.

**Figure 7:**
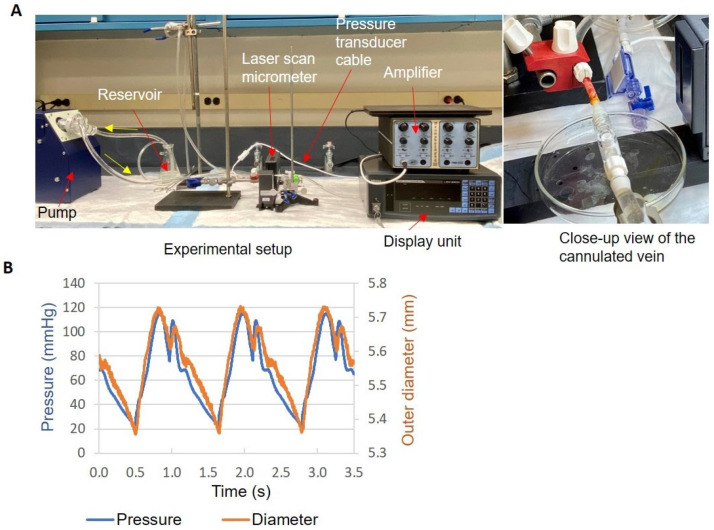
Human Vein Distensibility Assessment Using a Perfusion System. Distensibility of the veins were tested after VRS-AVF treatment with or without the step of light activation using the bench top perfusion system shown in Panel (**A**). Panel (**B**) illustrates an example of the measured pulsatile pressure and outer diameter tracings in three perfusion cycles. The blue line on the graph represents cyclical changes in the intraluminal pressure, and the red line represents the corresponding changes in the outer diameter of the veins detected by a laser scan micrometer.

A bench top perfusion system was used to test the distensibility of the veins after VRS-AVF treatment with or without the step of light activation (Figure 7A). The pulsatile pressure and outer diameter were stable as shown by three consecutive cycles in Figure 7B. The mean flow rate was 240 mL/min. The balloon distension pressure values were similar (8.8 ± 2.2 vs. 8.2 ± 2.7 atm for the veins with or without light activation, respectively), and the levels of over-dilatation were also similar (58 ± 12 vs. 55 ± 7% for the veins with or without light activation, respectively). The minimum, maximum, and pulse perfusion pressure values and the mean outer diameters during perfusion were similar (Table 1). The veins without light activation had a slightly larger percent outer diameter change during a pulsatile cycle and were slightly more distensible than the veins with light activation (Table 1), but the difference was not statistically significant (*p* = 0.25 and *p* = 0.23 for percent diameter change and distensibility, respectively).

### 2.5. Histological Comparison of Human Arm and Leg Veins

**Figure 8:**
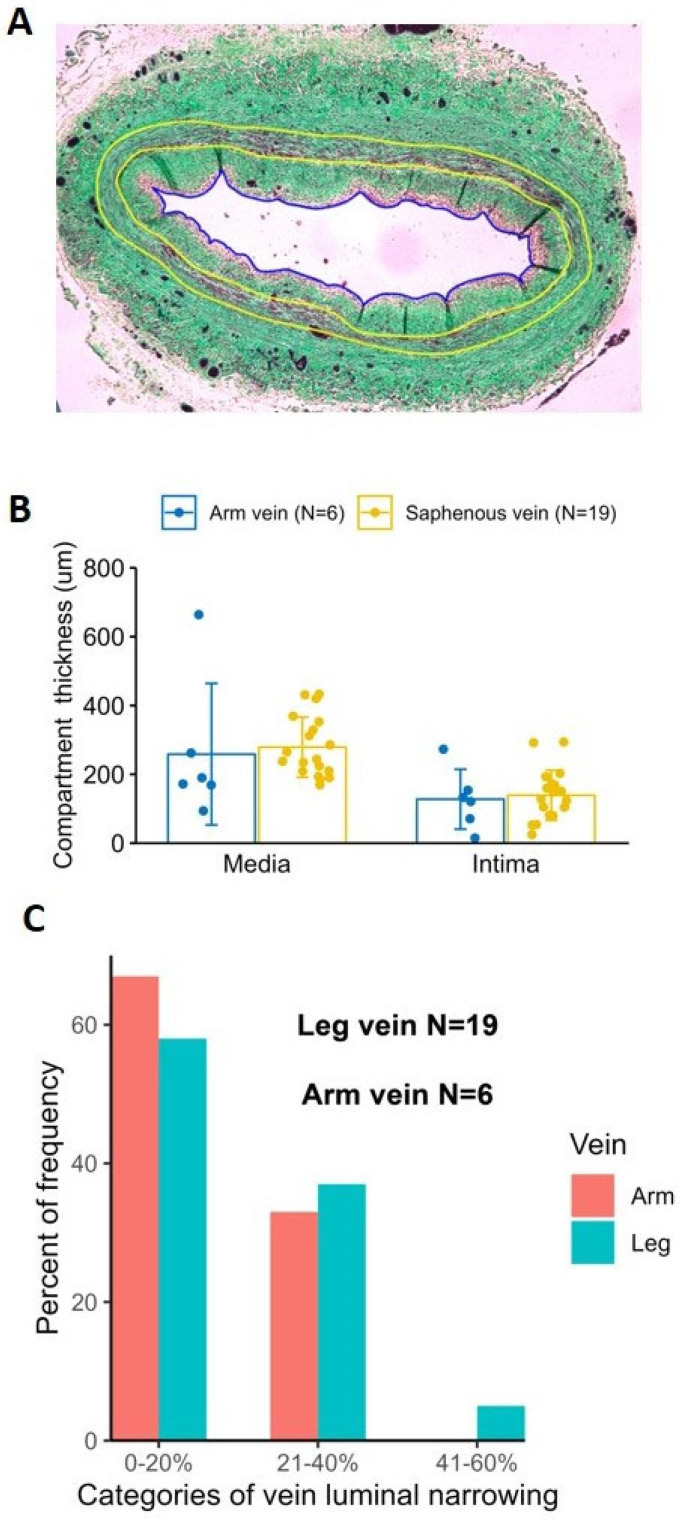
Human Saphenous Vein and Arm Vein Comparison. Panel (**A**) demonstrates a representative histology section of a human vein. The boundaries between the intima and media (IBL) and media and adventitia (EBL) are marked with yellow lines. The picture illustrates the method for extraction of lumen perimeter (blue line) and intima and media areas that were used to calculate mean wall thickness represented in Panel (**B**) and percent lumen narrowing due to intima formation represented in Panel (**C**).

The mean thickness of the intima and media compartments were not appreciably different between excised leg and arm vein segments (intima thickness: leg vein 139 ± 73 µm vs. arm vein 128 ± 89 µm, *p* = 0.75; media thickness: leg vein 279 ± 87 µm vs. arm vein 259 ± 256 µm, *p* = 0.73; Figure 8). Both vein types demonstrated a moderate degree of intimal hyperplasia, with no significant differences observed in the degree of intimal luminal narrowing (mean narrowing: leg vein 21 ± 10% vs. arm vein 19 ± 10%, *p* = 0.63) or the relative distribution of hyperplastic severity (Figure 8).

## 3. Discussion

The vascular ECM is an important regulator of both adaptive and pathological remodeling of the vascular wall in the cardiovascular system. ECM proteins, such as collagen fibers, elastin, and proteoglycans, are the major structural proteins of blood vessel walls. They convert mechanical stimuli generated by hemodynamic forces into biological signals via integrin-mediated mechanotransduction [14]. The integrity or reorganization of this local cellular environment is largely responsible for the adequate regulation of cell responses as the system adjusts to hemodynamic changes produced by gradually developing stenotic changes or acute surgical interventions, thereby determining the outcome of long-term vascular remodeling [13,23]. The VRS-AVF technology described in this paper targets the ECM of the vessel wall as a therapeutic principle by creating modifications of the ECM proteins during balloon-dilatation-initiated rearrangement to facilitate fistula maturation.

Insufficient dilatation of the venous outflow continues to be the primary failure point of fistula usability. Various approaches have been investigated to augment outward remodeling of the vein with limited success. Since endothelial cells are the key regulator of vascular structure and function, they have been used to broadly modulate the vascular biology and have been evaluated in the AVF setting. Perivascular placement of implants containing allogeneic endothelial cells around the surgical anastomotic sites reduced intimal hyperplasia at these sites in porcine AVF models [24]. For clinical use, allogeneic aortic endothelial cells were isolated from aortas of cadaver donors and cultured in a gelatin matrix [25]. Use of perivascular endothelial implants (Vascugel) around the surgical anastomotic sites and venous outflow sites in patients showed initial promise but failed to advance into clinical practice following a Phase I/II trial [25]. Some of the most interesting therapies have centered around the ECM and attempt to augment the remodeling in the days to weeks following fistula creation. Initial investigations utilized an angioplasty balloon to disrupt the matrix via repeated mechanical dilatation [26,27]. While several groups of practitioners endorsed this, it was never rigorously examined and fell out of favor due to lack of data [28]. Extension of matrix manipulation to the area of pharmacotherapeutics spawned the investigation of human pancreatic elastase, vonapanitase, applied topically at the time of AVF creation as a potential solution [29]. Examination within a well-structured Phase II clinical trial failed to show improvement [30]. In another study, vonapanitase also did not significantly improve primary patency of radiocephalic fistulas, although it was associated with increased secondary patency and use for hemodialysis [31]. Finally, a study with more than 600 patients demonstrated that vonapanitase treatment did not achieve clinical or statistical significance to meaningfully improve radiocephalic fistula surgical outcomes. It is important to note that the outcomes in the placebo group were better than those in historical controls [32]. Despite these failures, basic and translational science investigations have continued to point towards the ECM, particularly the three-dimensional structure of the collagen fibers, as the key component that dictates AVF maturation success or failure [33].

It has become clear that the ECM acts not as a passive framework but as a critical regulator of cell migration and proliferation. Therefore, the VRS-AVF treatment is an attractive approach for improved arterialization of the vein and a potentially vital adjunct to enhance AVF usability. Our hypothesis is that the newly formed covalent bonds within the ECM will support the balloon-dilatation-created lumen size and, concomitantly, allow the arterial-pressure-induced proliferative response of the cells to develop a thickened fistula wall via outward remodeling. Successful maturation is the clinical goal for the VRS-AVF treatment.

The effect of the photochemical reaction facilitating new covalent bond formation between oxidizable amino acids of the ECM proteins on AVF maturation using the 10-8-10 Dimer was first investigated in a well-studied rat AVF model [34]. In those experiments, femoral AVFs were created in young Wistar male rats. Treatment was applied immediately after the blood flow was restored and the arterial pressure dilated the femoral vein (which is achieved by the balloon inflation in the sheep model). In the rat model, a 10 µL drop of a 2 mg/mL solution of 10-8-10 Dimer in phosphate buffered saline (PBS) was placed at the anastomosis perivascularly and incubated for 5 min, giving the compound time to diffuse into the vascular wall from the adventitial side. Then, a 1 min illumination using 450 nm light initiated the photochemical reaction. Histological results from the rat model demonstrated significant outward remodeling at 4 weeks after treatment in comparison with controls treated only with saline solution. The increased lumen size was accompanied by reduced inflammation as measured by IL-6, MMP 2, and MMP 9 reduction and maintained cellular proliferation as measured by Ki67, a marker of cell proliferation. The results of this rat AVF experiment indicated that targeting the ECM proteins at the time of fistula creation reaffirms the ECM fibers and provides directionality to the proliferative process necessary for proper arterialization or venous wall maturation [34].

Most of the existing AVF animal models have been using arteries and veins with large starting diameter (femoral artery to femoral vein or carotid artery to jugular vein), being deeply embedded in protective layers of fascia and muscle fibers compared with the superficial veins most often used to create human hemodialysis fistulas [35]. These models are used to examine the anastomosis area or to study secondary failure of fistulas due to stenosis rather than investigating fistula maturation and growth, including the outflow segment. Selecting the right animal model to study human diseases and pathological processes is critical to gain translationally relevant information to determine therapeutic strategies. Florescu at al. introduced the sheep peripheral AVF model using superficial veins, but besides the surgical description, fistula maturation has not been investigated in this model [36]. Although it is surgically more demanding, and fistula maturation was not investigated, this sheep model is considered to be translationally more representative to test the use, performance, and vascular safety of our device for clinical use due to the location of the vein and the size of the animal.

Using this model, our data indicate that arterialization of the veins develops over time, and based on our monitoring of the proliferation marker Ki67, this process was significantly facilitated by the treatment around the first week following the surgery (Figure 2 and Figure 3). Interestingly, no intimal hyperplasia developed in the veins over time despite the use of an intravascular device, although acute albeit mild damage to the intima was noticed during histological investigation compared with control AVF. The lack of intimal hyperplasia development in the sheep model is different than what has been seen in other models using deeper and larger arteries and veins for AVF construction [35]. The original report on the surgical development of the sheep model by Florescu et al. did not include histomorphometric evaluation of the fistulas following surgery and maturation [36]. A dedicated study to systematically compare the potential contributing factors (such as initial vessel size, especially that of the supplying artery, the flow pattern, and pulse wave) to these histological differences in the models would be important in the future to aid optimal animal model selection. Nevertheless, the facilitated outward remodeling reproduces our previous findings in the rat model [34] and suggests that a more directionally controlled proliferative response of the vessel wall takes place following the photochemical treatment of the venous wall. The exact subcellular mechanism leading to the observed outward remodeling following photochemical treatment of the vein requires further investigation. However, it is conceivable that the new covalent bonds introduce structural changes to the balloon-distended ECM, which drive these processes by modifying the integrin signaling in response to the hemodynamic changes following fistula surgery. Previous studies, using the photochemical treatment with the 10-8-10 Dimer, found altered structural appearance of ECM proteins and reduced ECM protein fragmentation, suggesting that the treatment provides lasting structural changes and resistance against matrix degrading enzymes [14]. 

A shortcoming of any animal model used to study venous wall morphology and remodeling is the significant difference in vessel wall thickness of animal veins compared with human veins, especially those from patients who may become candidates for fistula surgery. Preexisting abnormalities, inflammation, cell proliferation, and even calcification have been described in human veins to be used for fistula creation [37]. The average size of the human vein wall, intima and media included, can be approximately two–three times thicker than the sheep cephalic vein wall. Another hindsight of the sheep model is that the cephalic veins of the sheep still have a relatively large diameter, and using the VRS-AVF device created only minimal over-dilatation of the venous wall from the baseline diameter. This has likely diminished the stretch-induced activation of downstream proliferative signals affecting the medial SMCs and hindered our investigation of potential damage to the venous wall by balloon dilatation. Understanding the consequences of these morphological differences is important for the development of a safe and efficacious treatment for human use. Therefore, we turned to investigating human veins, freshly excised during amputation or in preparation for bypass surgery, to investigate the safety of lumen dilatation by VRS-AVF.

Capitalizing on the high frequency of lower extremity operations in most vascular surgery practices, the current experiments mostly utilize excised segments of human saphenous veins to explore the system. Our results suggest that human veins can be dilated safely, without major structural damage up to 66% of their original diameter (Figure 5 and Figure 6). Along with histological investigation, cannulated- and balloon-expanded vein segments have also been exposed to pulsatile pressure to detect changes in distensibility (Figure 7). These measurements did not detect any adverse effect by the treatment on the vein biomechanics, maybe some increase in the ability to resist large arterial pressure changes, but these differences were not statistically significant. Although limited in overall sample numbers, there was no fundamental difference identified that would affect the safety of the treatment during upper extremity surgeries comparing arm veins to leg veins (Figure 8).

In conclusion, VRS-AVF offers a novel, safe, and promising drug device combination treatment for human veins applied at the time of fistula creation surgery. The structural changes of the ECM induced by the VRS-AVF treatment of the vein could potentially accelerate the maturation process of the fistula following surgery and may allow inclusion of suboptimal veins to be used successfully in fistula creation. These experimental results support clinical testing of the safety and efficacy of the VRS-AVF technology for the benefit of patients requiring vascular access for hemodialysis.

## 4. Materials and Methods

### 4.1. VRS-AVF Treatment

The treatment with the VRS-AVF device delivers a photoactivatable small molecule, 10-8-10 Dimer, locally to the vein (Figure 1). The VRS-AVF device is a unique endovascular device, as it carries a light fiber inside the balloon and a water-soluble 10-8-10 Dimer molecule as a coating on the external surface of the balloon. The tip of the balloon is also modified to be olive-shaped to assure an atraumatic insertion of the device into the vein without the need of fluoroscopy or ultrasound imaging. Upon insertion of the 100 mm length balloon into the vein, the balloon is inflated for 60 s, allowing the 10-8-10 Dimer molecules to diffuse into the vein wall. Light activation of the molecule with a 450 nm light, at maintained balloon distension of the vein for an additional 60 s, results in new covalent bond formation among ECM proteins of the vein wall to retain the size of the balloon. Creating this reinforced scaffold around the dilatated lumen aims to mitigate the hemodynamic stress.

### 4.2. Sheep Model of AVF Using Superficial Veins of the Forelimbs

To test the vascular safety of the VRS-AVF device in a translationally relevant animal model, a previously described large animal model of AVF using superficial veins of the forelimbs in sheep was used [36]. The longer accessible outflow vein segment allows investigation of the treatment effect on both the proximal vein, closer to the anastomosis, and the distal vein segment as the future cannulation zone. Studies were performed at Covance Laboratories Inc. (San Carlos, CA, USA). Nine 90–100 kg sheep (crossbred and purebred) were obtained for these studies. AVFs were created between the radial artery and cephalic vein of the forelimbs in an end-to-side configuration, as previously described [36]. The one-week survival study consisted of three sheep receiving bilateral AVF creation, and the four-week survival study consisted of six sheep with twelve AVFs created. The VRS-AVF catheter (Figure 1) was used to treat the vein prior to fistula creation on one limb, and the vein of the contralateral limb of the same animals remained untreated to evaluate the vascular effects caused by the VRS-AVF catheter treatment compared with AVF creation surgery alone.

### 4.3. VRS-AVF Procedural Steps during Fistula Creation Surgery

**Figure 9:**
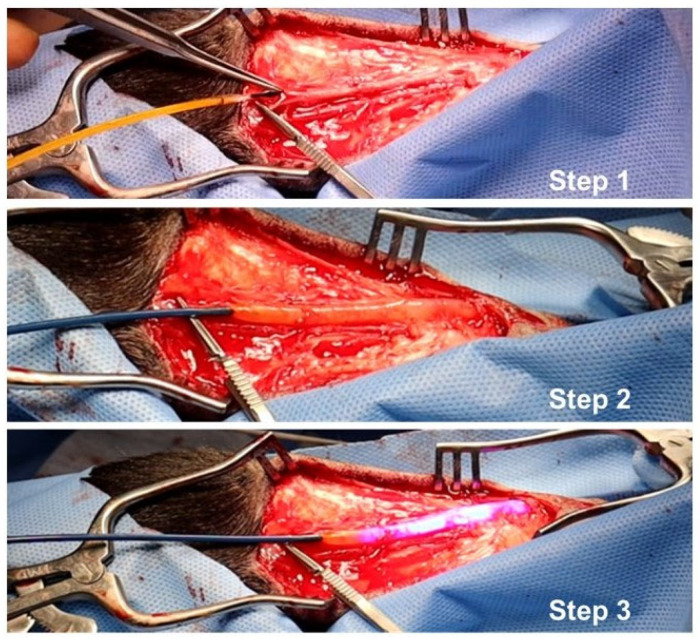
Photographic Depiction of the VRS-AVF Treatment Steps during Sheep Fistula Creation Surgery. Step 1 (Upper Panel): The device coated with the 10-8-10 Dimer is introduced into the vein without fluoroscopy. Step 2 (Middle Panel): The balloon is inflated inside the vein to dilate the vein lumen. Step 3 (Lower Panel): The light treatment activates the drug in the venous wall creating covalent linkage of the extracellular matrix proteins.

Treatment of the vein prior to the anastomosis creation surgery consisted of three major steps (Figure 9). During Step 1, the sheep cephalic vein and radial artery were exteriorized. The 100 mm balloon, coated with 10-8-10 Dimer, was introduced to the vein intraluminally without fluoroscopy. The olive-shaped tip of the balloon helped guide the atraumatic insertion of the device and its tracking through the vein. After insertion, the balloon was inflated to dilate the vein lumen to a desired size for one minute (5 mm in the sheep study) (Step 2) when the 10-8-10 Dimer diffused into the vein wall. The light fiber was then illuminated during the second minute of the inflation (Step 3). The light treatment initiates a photochemical reaction with 10-8-10 Dimer in the venous wall facilitating new covalent bond formation among oxidizable amino acids of the ECM proteins. Ultrasound imaging was used to monitor the fistula patency post-operatively and weekly for the duration of the four-week study. Patency of the fistulas was also confirmed with a fistulogram at the end of the study prior to necropsy. During necropsy, the veins were formalin-fixed by pressurized perfusion prior to dissection. The AVF and attached proximal and distal vasculature were excised en bloc and immersion-fixed in 10% neutral buffered formalin.

### 4.4. Morphometric Analysis and Immunohistochemistry Staining

For the safety evaluation and morphometric analysis, the formalin-fixed samples were paraffin-embedded (FFPE blocks), sectioned at a 5 µm thickness and stained with hematoxylin and eosin (H&E). All layers of the artery and vein were evaluated, including the endothelial layer, intimal layer, internal elastic lamina, tunica media, external elastic lamina, and adventitia. In the venous cross sections, boundary lines were created during the imaging process to separate the intimal, medial, and adventitial areas. Since neointima formation was rarely seen, the media area was calculated by subtracting the lumen area from the area encircled by the external boundary line. Due to the deformation of the histological cross section, the lumen area was not used to calculate lumen diameter directly; the length of the perimeter of the lumen was instead measured to calculate the lumen diameter. The average thickness of the media was calculated using the media area and lumen diameter. In addition, immunohistochemistry was also performed to stain for a proliferation marker, Ki67 (clone D3B5, product number 12202, Cell Signaling) and smooth muscle cell marker, alpha actin (SMA) for morphological confirmation of the media (clone D4K9N, product number 19245, Cell Signaling) [21]. Slides were stained on a Leica Bond RX autostainer (Leica Biosystems, Salt Lake City, UT, USA). All sectioning and staining were performed by Huntsman Cancer Institute Biorepository and Molecular Pathology Core laboratory (Salt Lake City, UT, USA). Digital brightfield images were captured using a 10× objective with identical light and camera capture settings on a Zeiss Axio Scan.Z1 microscope located on the University of Utah campus, Cell Imaging Core. Digital images were analyzed using off-the-self digital image analysis software (MIPAR v4.2 Columbus, OH) that was used to determine the quantity of Ki67-positive nuclei. All images were evaluated in a similar fashion. The algorithm determined Ki67-positive nuclei by using both color and size as a determining factor. After the initial automatic determination of Ki67-positive and normal nuclei, a manual cleanup step was completed to ensure the tunica media as the area of interest by removing all artifacts and features not belonging to the tunica media. Nuclei counts, both Ki67 positive and normal, were tallied by the software with the proliferation index (PI), percentage of proliferating cells, as described in Equation (1).

(1) $$PI=\frac{\#\;of\;Ki67-Positive\;Nuclei}{Total\;\#\;of\;Nuclei}\times 100$$

### 4.5. Human Vein Studies

Human saphenous veins harvested from amputated legs or cephalic vein leftover from lower-extremity bypass grafts were used for damage evaluation, drug delivery, biomechanical assessment, and morphometric analysis. Vein samples had various lengths, ranging from 0.5 cm to 10 cm, and the internal diameter was calculated on the basis of circumference measurement, as described by Munger and colleagues [38]. After removing excess perivascular tissue, a 2 mm long segment of vein was cut open, gently flattened between two glass microscope slides and the length (circumference) measured by a digital caliper. The diameter of the drug-coated balloon and the inflation pressure were chosen to achieve various levels of vessel over-dilatation ranging from ~25% to ~100%. The length of the balloon used in this set of experiments was 4 cm. Veins were dilatated for one minute before the 1 min light activation with the balloon inflated (Figure 5). For the purpose of drug delivery testing, the vein was immediately frozen in liquid nitrogen.

Using a bench top perfusion system, 12 vein segments after VRS-AVF treatment with or without light activation were cannulated to evaluate the effect of crosslinking between the drug and ECM proteins on distensibility (Figure 7). A pulsatile blood pump (model 1421, Harvard Apparatus, Holliston, MA, USA) created the pressure pulse, which was measured by a pressure transducer (BLPR2) and a four-channel amplifier (TBM4M, both from World Precision Instruments, LLC, Sarasota, FL, USA). A Mitutoyo laser scan micrometer (LSM-503S with the LSM-6200 display unit, MSI Viking, Duncan, SC, Canada) measured the vein outer diameter at the middle. The length of the vein used in this testing was approximately 3 cm. The pressure and diameter data were collected simultaneously every 2.5 milliseconds through a multifunction I/O Device (USB-6001) with LabVIEW SignalExpress software (Version 5.1; National Instruments, Austin, TX, USA) after at least 5 min of pump running. Calcium-free PBS at room temperature was used as the perfusion solution. Distensibility was calculated as described in Equation (2).

(2) $$Distensibility=\frac{A_{max}-A_{min}}{A_{min}\cdot ∆P}$$

where $A_{max}$ and $A_{min}$ are the maximal and minimal areas of the vein within a cycle calculated from the outer diameter, respectively, and ∆P is the pulse pressure. If leakage occurred during perfusion, the vein was classified as damaged and excluded from distensibility calculation.

As in the sheep samples, the human vein samples were also fixed in formalin, embedded in paraffin, sectioned at a 5 µm thickness, and stained with Masson’s Trichrome. The length of the perimeter of the lumen was measured to calculate the lumen diameter ($D_{lumen}$) or radius ($R_{lumen}$). The intima area, $A_{intima}$, was obtained by subtracting the lumen area from the area encircled by internal boundary line (*A_IBL_*) (Figure 8). The media area, $A_{media}$, was obtained by subtracting *A_IBL_* from the area encircled by external boundary line (*A_EBL_*). The radii of IBL and EBL were calculated as described in Equations (3) and (4) below.

(3) $$R_{IBL}=\sqrt{\frac{A_{intima}}{\pi }+R_{lumen}^{2}},$$

(4) $$R_{EBL}=\sqrt{\frac{A_{media}}{\pi }+R_{IBL}^{2}}.$$

The average thickness of the intima was calculated as $R_{IBL}-R_{lumen}$, and the average thickness of the media was calculated as $R_{EBL}-R_{IBL}$. To quantify the lumen narrowing due to intima formation, the percent lumen narrowing was defined in Equation (5).

(5) $$\frac{A_{intima}}{A_{intima}+A_{lumen}}\times 100\%,$$

where $A_{lumen}=\pi {\cdot R}_{lumen}^{2}$. The percent lumen narrowing was categorized as 0–20%, 21–40%, 41–60%, 61–80%, and 81–100% [39]. Of note, the IBL and EBL correspond to the boundary of the innermost and outermost smooth muscle cells, respectively, for the vein samples.

### 4.6. Fluorescent Images of Treated Veins to Confirm Drug Delivery

10-8-10 Dimer penetration was quantified on histological sections relying on the fluorescent detectability of the 10-8-10 Dimer molecule using the Zen2.5 lite software (Zen Imaging Software, Carl-Zeiss, Jena, Germany) (Figure 4). Data were collected from four different representative profiles, with 0°, 90°, 180°, and 270° being the preferred locations for fluorescent intensity determination. To obtain unobscured profiles at these positions, sampling was adjusted as needed.

### 4.7. Statistical Analysis

Data were presented as mean ± standard deviation. Data from multiple sections of the same vein sample were averaged, and the average values were used in further analyses. The associations between sheep vein morphologic parameters and treatment and post-creation time were examined by a linear mixed-effect model with the animal as the random factor. The significance of the comparison between VRS-AVF treated and control sheep AVFs was then evaluated by paired t-tests. The wall thicknesses of sheep and human arm veins were compared using the Mann–Whitney Rank Sum Test. Other comparisons were performed using t-tests. All statistical analyses were performed in R (Version 4.4.2; A Language and Environment for Statistical Computing, R Core Team 2022; R Foundation for Statistical Computing, Vienna, Austria (www.R-project.org)) or Sigmaplot (Version 11.0, Systat Software Inc., Chicago, IL, USA). A *p*-value < 0.05 was considered statistically significant.

## Figures and Tables

**Table 1:** Human Vein Bench Top Biomechanics Test Conditions and Results.

Light Activation	Pr Min (mmHg)	Pr Max (mmHg)	Pulse Pr (mmHg)	Mean D (mm)	D Change (%)	Distensibility (10^−3^/mmHg)
Yes	20 ± 5	110 ± 6	91 ± 2	5.33 ± 0.55	3.9 ± 1.9	0.87 ± 0.41
No	18 ± 1	108 ± 2	90 ± 1	5.39 ± 0.58	5.1 ± 1.7	1.17 ± 0.40

Pr: intraluminal perfusion pressure; D: vein outer diameter. Each condition had six vein segments.

## Data Availability

The data presented in this study are available on request from the corresponding authors (Scott A. Berceli or Katalin Kauser).
